# Combined inhibition of XIAP and autophagy induces apoptosis and differentiation in acute myeloid leukaemia

**DOI:** 10.1111/jcmm.17765

**Published:** 2023-05-08

**Authors:** Ziyang Huang, Jifan Zhou, Yinyan Jiang, Yixiang Han, Xiaofang Wang, Fanfan Li, Songfu Jiang, Kang Yu, Shenghui Zhang

**Affiliations:** ^1^ Department of Hematology The First Affiliated Hospital of Wenzhou Medical University Wenzhou Zhejiang China; ^2^ Institute of Hematology Wenzhou Medical University Wenzhou Zhejiang China; ^3^ Wenzhou Key Laboratory of Hematology Wenzhou Zhejiang China; ^4^ Central Laboratory The First Affiliated Hospital of Wenzhou Medical University Wenzhou Zhejiang China; ^5^ Laboratory Animal Center The First Affiliated Hospital of Wenzhou Medical University Wenzhou Zhejiang China

**Keywords:** acute myeloid leukaemia, apoptosis, autophagy, differentiation, XIAP

## Abstract

Perturbations in autophagy, apoptosis and differentiation have greatly affected the progression and therapy of acute myeloid leukaemia (AML). The role of X‐linked inhibitor of apoptosis (XIAP)‐related autophagy remains unclear in AML therapeutics. Here, we found that XIAP was highly expressed and associated with poor overall survival in patients with AML. Furthermore, pharmacologic inhibition of XIAP using birinapant or XIAP knockdown via siRNA impaired the proliferation and clonogenic capacity by inducing autophagy and apoptosis in AML cells. Intriguingly, birinapant‐induced cell death was aggravated in combination with ATG5 siRNA or an autophagy inhibitor spautin‐1, suggesting that autophagy may be a pro‐survival signalling. Spautin‐1 further enhanced the ROS level and myeloid differentiation in THP‐1 cells treated with birinapant. The mechanism analysis showed that XIAP interacted with MDM2 and p53, and XIAP inhibition notably downregulated p53, substantially increased the AMPKα1 phosphorylation and downregulated the mTOR phosphorylation. Combined treatment using birinapant and chloroquine significantly retarded AML progression in both a subcutaneous xenograft model injected with HEL cells and an orthotopic xenograft model injected intravenously with C1498 cells. Collectively, our data suggested that XIAP inhibition can induce autophagy, apoptosis and differentiation, and combined inhibition of XIAP and autophagy may be a promising therapeutic strategy for AML.

## 1 INTRODUCTION

Acute myeloid leukaemia (AML) is an aggressive disease in which immature blood cells develop abnormally and grow uncontrollably. Despite decade‐long efforts on basic and clinical research, the current mainstay of treatment is still remission‐inducing chemotherapy with cytarabine and anthracycline.^1^ Recently, despite allogeneic haematopoietic stem cell transplantation (allo‐HSCT) is more applied in clinical, the patients still have a relatively low 5‐year survival rate, especially the elderly patients who are considered unsuitable for standard induction chemotherapy.^2^

X‐chromosome‐linked inhibitor of apoptosis protein (XIAP), located on X chromosome at Xq24‐25 in human, is one of the IAP family members. XIAP contains three N‐terminal baculoviral IAP repeat (BIR) domains, a ubiquitin‐associated (UBA) domain and a C‐terminal RING finger domain. Mounting evidence demonstrates that XIAP promotes the growth, invasion, and metastasis of malignant cells, confers resistance to certain chemotherapeutic drugs, and associates with poor outcome in a variety of cancers.^3^, ^4^ Overexpression of XIAP is frequently observed in human cancers and may contribute to tumorigenesis by evading cancer cell apoptosis.^5^, ^6^ As for AML, multiple reports have regarded XIAP as a unfavourable gene through a variety of signal pathways, including caspase pathway^7^, ^8^ and NF‐kB pathway,^9^ and inhibition of XIAP can sensitize AML cells to tumour necrosis factor‐related apoptosis‐inducing ligand (TRAIL).^10^, ^11^ Recently, several studies also show that XIAP inhibitors can increase the efficacy of BCL2 inhibitors^12^ or affect AML myelomonocytic differentiation.^13^ Besides, recent advances have revealed that XIAP is involved in the regulation of autophagy in multiple cells.^14^, ^15^ However, the role of XIAP on autophagy is still contradictory, some works find XIAP may suppress autophagy^14^ and others show XIAP can induce autophagy.^16^

Autophagy is a conserved catabolic process whose role in cancer is complex, as it constrains tumour initiation by maintaining cellular and genomic integrity whereas promotes tumour progression in established tumours by favouring cancer cell survival.^17^ As for AML, autophagy is a double‐edged sword, with increasing evidence that autophagy plays a critical role in AML tumorigenesis and anti‐leukaemic responses.^18^ The oncogenic fusion proteins and genetic mutations, such as MLL‐AF9, FLT3‐ITD and mutated NPM1, can activate autophagy to promote proliferation in AML initiation and progression.^19^, ^20^, ^21^ Autophagy is essential for the degradation of PML‐RARα fusion protein when acute promyelocytic leukaemia (APL) cells are treated with all‐trans retinoic acid (ATRA) or arsenic trioxide.^22^ Deficient of aberrant differentiation is a hallmark of AML. At present, the relationship between autophagy and differentiation is mainly studied in the area of APL. Two studies have showed that autophagy exhibits a crucial role in ATRA‐induced differentiation therapy.^23^, ^24^ Accumulating evidence has highlighted that modulation of autophagy provides therapeutically beneficial effects for a certain subgroup of AML patients.

Here, we investigate the detailed role of XIAP on autophagy regulation in AML and have found that XIAP inhibition by pharmacologic or genetic knockdown can inhibit the proliferation and clonogenic capacity of AML cells, and promote their apoptosis, autophagy and differentiation. Spautin‐1, an inhibitor of autophagy, can enhance the level of reactive oxygen species (ROS) and myeloid differentiation of AML cells induced by XIAP inhibition. Two mouse AML models also reveal that the combination with an autophagy inhibitor chloroquine (CQ) can enhance the inhibitory effect of a XIAP inhibitor birinapant on AML progression.

## 2 MATERIALS AND METHODS

### 2.1 Enrolled patients and bone marrow samples

Human AML cell lines THP‐1, HEL, HL‐60, U937 and mouse AML cell line C1498 were purchased from the American Type Culture Collection (ATCC, USA) and cultured in RPMI‐1640 medium supplemented with 10% foetal bovine serum (FBS; both from Gibco, USA) at 37°C in 5% CO_2_. A total of 63 newly diagnosed AML patients including 9 APL and 54 Non‐M3 AML, which consists of 29 males and 25 females with a median age of 52 years old (range: 15–84), were enrolled at Department of Haematology in the First Affiliated Hospital of Wenzhou Medical University (Table S1). All patients with AML were diagnosed and classified according to the 2016 WHO Classification for AML. 19 healthy donors matched for age and gender, including 11 males and 8 females, were enrolled as controls. Bone marrow mononuclear cells (BMMNCs) were isolated from the bone marrow aspirates by Ficoll–Hypaque density‐gradient centrifugation (Haoyang Institute of Biotechnology, China) and further positively selected for CD34^+^ cells using CD34^+^ magnetic bead cell sorting (Miltenyi Biotec, Germany). These CD34^+^ cells from AML patients were regarded as primary blast cells, which were cultured in RPMI‐1640 medium containing 20% FBS, and then maintained at 37°C in 5% CO_2_. Overall survival (OS) was defined as the time from enrollment to death from any cause. Patients alive or lost to follow‐up are censored. The Institutional Review Board of the First Affiliated Hospital of Wenzhou Medical University approved this study, and all participants signed informed consent in accordance with the Declaration of Helsinki protocol.

### 2.2 Quantitative real‐time PCR

AML cells were transfected with XIAP, ATG5 or AMPKα1 siRNAs (GenePharma) by Lipofectamine® RNAiMAX Reagent (Invitrogen™) and then identified by analysis of mRNA and protein expression. The total RNA was extracted from cells using TRIzol™ reagent (Life Technologies) and reverse transcribed into cDNA, and then cDNA was amplified and quantified using TB Green® Premix Ex Taq II (Takara, Dalian, China) with primer pairs on a 7500 Real‐Time PCR System (Applied Biosystems®). All results were standardized against GAPDH endogenous control, and the relative quantity (RQ) was computed as 2^−ΔΔCq^. Specific primer and siRNA sequences were showed in Tables S2 and S3.

### 2.3 Western blot and co‐immunoprecipitation (Co‐IP) analyses

The cells were collected, and lysed in RIPA lysis buffer with PMSF and protease inhibitor cocktail (Beyotime Biotechnology, Shanghai, China). Then, these protein samples were boiled and further subjected to western blot analysis with different antibodies, including p53 (#2527), SQSTM1/p62 (#8025), p‐AMPKα1 (#4184), AMPKα (#2532), mTOR (#4517), p‐mTOR (#5536), PARP (#9542), LC3A/B (#4108), XIAP (#14334), MDM2 (#86934) and GAPDH (#2118; all from Cell Signalling Technology, USA), at 1:1000 dilution overnight at 4°C, respectively. For Co‐IP, the cells were lysed in RIPA lysis buffer with PMSF on ice for 30 min, and a BCA protein assay kit was used to determined protein concentration. To eliminate non‐specific proteins, 500 μg protein lysate per sample was incubated with 20 μL protein A/G‐agarose beads and 1 μg mouse‐control IgG for 1 h, and then centrifugated at 2400 × g for 5 min. The A/G‐agarose beads with non‐specific proteins were discarded while the supernatants were collected and incubated with beads coated with 10 μg/mL mouse‐source anti‐MDM2 or 1 μg control IgG overnight at 4°C. After washing three times by lysis buffer, these bead‐protein mixtures were boiled and subjected to western blot analyses with antibodies including mouse‐source anti‐MDM2 (#AF208, Affinity, at 1/1000 dilution), rabbit‐source anti‐p53 (#2527, CST, at 1/1000 dilution), anti‐XIAP (#14334, CST, at 1/1000 dilution), respectively. A FluorChem E with AlphaView SA software (Version: 3.4.0.0) (both from ProteinSimple) was used to scan and analyse the optical densities of the bands.

### 2.4 Cell viability assay and Colony formation assay

Cell viability was detected by cell counting kit‐8 (CCK‐8; MedChemExpress, Shanghai, China) and cell number was counted manually. The absorbance was read at 450 nm using a microplate reader (Synergy H1; BioTek).

Following the directions of the manufacturer, MethoCult™ H4034 optimum (STEMCELL™ Technologies) was used to assess the clonogenic capacity. Visible colonies comprised of at least 60 cells were counted and photographed under a microscope (BX51; Olympus).

### 2.5 Apoptosis assay, myeloid cell differentiation assay and ROS level analysis

Apoptosis was determined using the Annexin V‐FITC/PI apoptosis kit (MultiSciences, Hangzhou, China) on a Navios Flow Cytometer (Beckman coulter). After pre‐treatment with 10 μM spautin‐1 for 1 h, AML cells were treatment with or without birinapant for 48 h. At 6 hours after treatment, 5 × 10^5^ THP‐1 cells were collected and incubated with DCFH‐DA for 20 min at 37°C using a Reactive Oxygen Species Assay Kit (Beyotime Biotechnology), and then, the intracellular ROS levels were measured on a flow cytometry. At 96 h after treatment, another 5 × 10^5^ THP‐1 cells were collected and stained with anti‐CD11b‐APC‐A750, anti‐CD14‐PC7 and anti‐CD15‐PC5.5 (all from Beckman coulter), and then, the differentiation of these cells was determined on a flow cytometer. All data were analysed with FlowJo v10 (BD, USA).

### 2.6 Animal models

Female 4–6 weeks old NOD‐SCID mice and Female 4–6 weeks old C57BL/6 mice were purchased from Charles River (Beijing, China) and bred under SPF conditions in the Laboratory Animal Center of the First Affiliated Hospital of Wenzhou Medical University. All of mouse experiments have been approved by the Laboratory Animal Ethics Committee of the First Affiliated Hospital of Wenzhou Medical University and have been performed in accordance with relevant institutions and national guidelines and regulations.

Cyclophosphamide (Macklin, Shanghai, China) at 80 mg/kg was administered intraperitoneally (i.p.) before tumour inoculation, and then, a total of 5 × 10^6^ HEL cells were subcutaneously injected into NOD/SCID mice. When the tumour size reached about 150–200 mm^3^, mice were randomized into four groups (*N* = 9 mice per group). The vehicle group was given saline, and the treatment groups were injected with birinapant three times per week (40 mg/kg, i.p.) or CQ three times per week (50 mg/kg, i.p.) (Sigma‐Aldrich) or both agents, respectively. Tumour length and width were measured every 2 days, and the volume was calculated using the formula: volume = length × width^2^ × 0.5236. All mice were sacrificed under anaesthesia with isoflurane (Sigma‐Aldrich) on 14 days after treatment with birinapant or CQ.

C57BL/6 mice were injected intravenously with 1 × 10^6^ C1498 cells to establish orthotopic AML xenograft model (*N* = 16 mice per group). Four mice per group were sacrificed under anaesthesia with isoflurane on 15 days after tumour inoculation for analysis. Tumour infiltration in peripheral blood, bone marrow, and liver tissues were determined by Wright‐Giemsa stain, immunohistochemistry and flow cytometry as following. Another 12 mice per group were bred and their death time was recorded for survival analysis.

### 2.7 Statistical analysis

Two‐tailed unpaired Student's *t*‐test with 95% confidence interval (CI) was applied to compare the mean of two independent group. One‐way anova test with Dunnett's method for multiple comparisons with 95% CI was applied to compare the data involving two or more test groups and a control group. One‐way anova with Tukey test was applied to compare among up to three groups. *p* value <0.05 represents a statistically significant difference. All experiments were conducted with three replicates, unless stated. All statistical analyses were performed using GraphPad Prism 6.0 (GraphPad software, CA, USA).

## 3 RESULTS

### 3.1 XIAP is overexpressed and correlates with poor prognosis in AML

**FIGURE 1:**
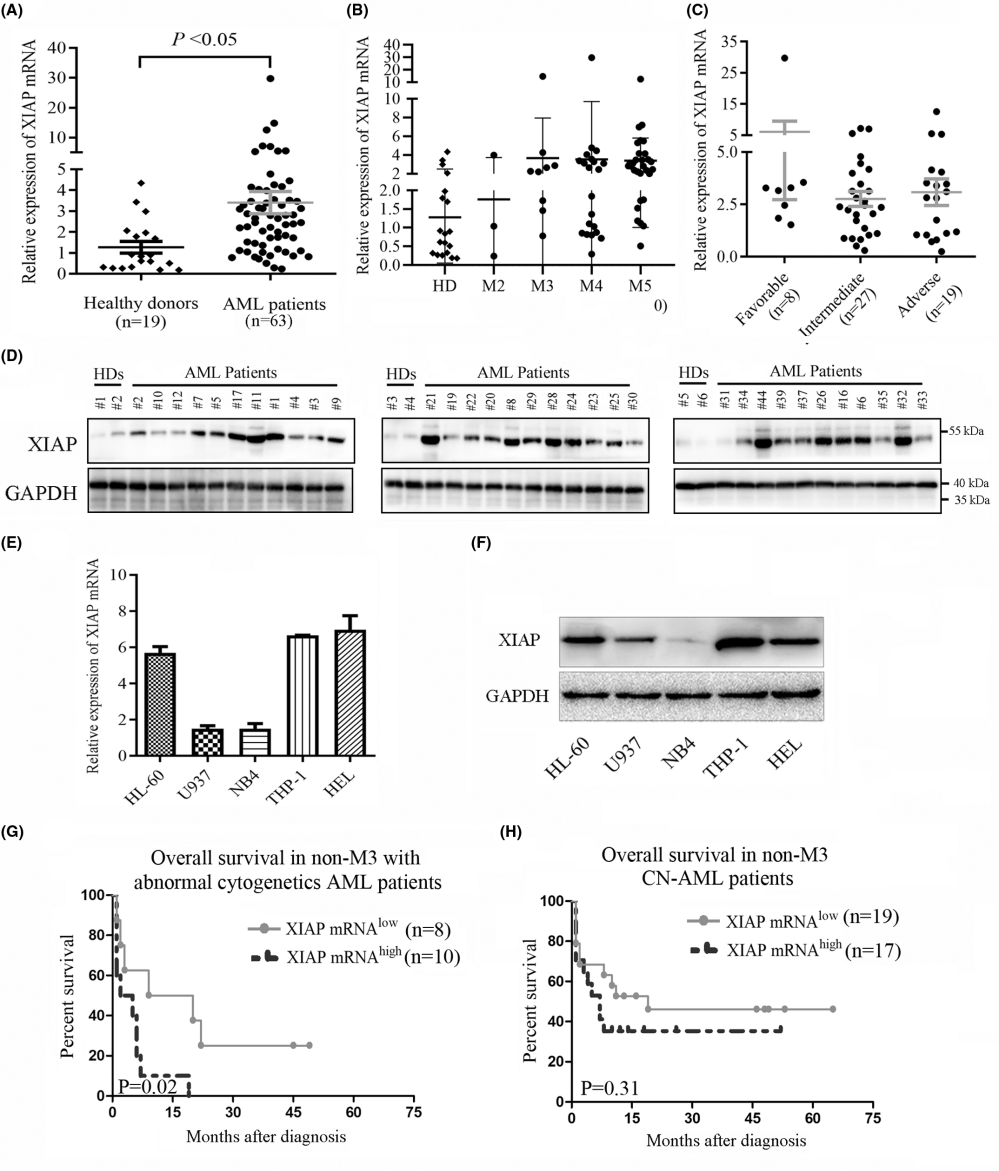
XIAP is elevated and grants poor prognosis in AML. (A, B) Relative expression of XIAP mRNA was determined in BMNCs from healthy donors (*N* = 19) and newly diagnosed AML patients (*N* = 63), which was further exhibited in different FAB subtypes. (C) Relative level of XIAP mRNA was showed according to risk status based on validated cytogenetics and molecular abnormalities in patients with Non‐M3 AML. (D) The XIAP protein expression in BMMNCs from healthy donors (*N* = 6) and newly diagnosed AML patients (*N* = 33) was shown. (E, F) Relative expression of XIAP mRNA and protein was showed in several AML cell lines. (G, H) The Kaplan–Meier analysis with a log‐rank statistical test showed that the cytogenetically abnormal non‐M3 AML patients with higher XIAP expression had a shorter overall survival than those with lower XIAP expression, but not in cytogenetically normal non‐M3 patients. Data are expressed as mean ± SD. Ns, * and ** represent *p* > 0.05, *p* < 0.05 and *p* < 0.01, respectively.

XIAP has been reported to be highly expressed in a variety of malignant tumours and closely related to their occurrence, development and prognosis.^25^, ^26^ Herein, we found that mRNA expression of XIAP was evidently higher in BMMNCs isolated from the newly diagnosed AML patients than those from healthy donors (Figure 1A). But there was no statistical difference in the level of XIAP mRNA among the different FAB types (Figure 1B) or risk groups according to European leukemiaNet 2017 risk stratification (Figure 1C). There were also higher expression of XIAP mRNA in CD34^+^ cells isolated from patients than those from healthy donors (Figure S1). Compared to healthy donors, higher expression of XIAP protein was also observed in AML patients (Figure 1D and Figure S1B). Moreover, high expression of XIAP mRNA and protein was exhibited in four AML cell lines HL‐60, U937, THP‐1 and HEL (Figure 1E,F). Non‐M3 patients with higher expression of XIAP had a worse overall survival (OS) than those with lower expression of XIAP (Figure S2A), especially in those patients with abnormal cytogenetics (Figure 1G) but not with normal cytogenetics (Figure 1H). All AML patients or M3 patients with higher expression of XIAP had no difference in OS compared with those patients with lower expression of XIAP (Figure S2B,C). The comparison of clinical characteristics in patients with lower and higher XIAP expression was shown in Table S4. Compared to patients with lower XIAP expression, those with higher expression of XIAP had a trend towards harbouring higher WT1 expression (*p* = 0.082). But there was no difference in other variables including age, haemoglobin levels and platelet counts between these two groups.

In univariate analysis (Table S5), the independent poor risk factors for OS were age older than 60 years, unfavourable karyotype, higher XIAP expression and TET2 mutation. We further analysed the meaning of XIAP expression using a multivariate analysis that includes variables significantly associated with clinical outcome. Age, higher XIAP expression and unfavourable karyotype were still independent poor prognostic factors for OS (Table S6).

### 3.2 XIAP facilitates proliferation and clonogenic capacity in AML cells

**FIGURE 2:**
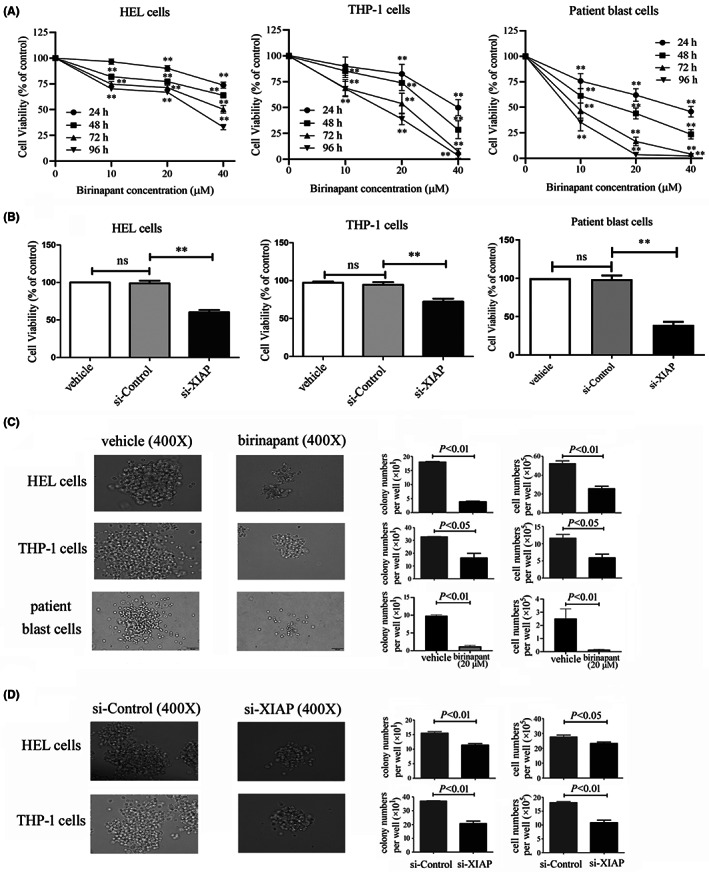
XIAP inhibition impairs cell viability and clonogenic capacity in AML cells. (A) After treatment with various concentration of birinapant for 0, 24, 48, 72 and 96 h, the cell viability of two AML cell lines HEL and THP‐1 as well as patient blast cells was measured by CCK‐8 assay. (B) After transfection with si‐Control or si‐XIAP‐3 for 48 h, the cell viability of HEL and THP‐1 cells as well as patient blast cells was determined by CCK‐8 assay. (C) The colonies were observed under a microscopy in HEL and THP‐1 cells as well as patients blast cells after treatment with or without birinapant at 20 μM for 8 days or 14 days. (D) The colonies were observed in HEL and THP‐1 cells after transfection with si‐Control or si‐XIAP‐3 for 8 days. Representative images were shown, and scale bar represents 50 μm. Results are expressed as mean ± SD representing at least three independent experiments. Ns and ** represent *p* > 0.05 and *p* < 0.01, respectively.

To identify the functional characterisation of XIAP in AML cells, siRNAs were designed to knockdown XIAP (Figure S3A,B) and birinapant, a Smac‐mimetic IAP‐antagonist was used to inhibit the expression of XIAP. We found that birinapant evidently decreased the cell viability of HEL and THP‐1 cells as well as patient blast cells in a time‐ and dose‐dependent manner (Figure 2A). XIAP siRNA also markedly reduced the cell viability of these cells (Figure 2B). Colony formation assays demonstrated that XIAP inhibition using birinapant or siRNA dramatically impaired the clonogenic capacity of AML cells with small colony size, decreased colony number and total cell number (Figure 2C,D). Similarly to cell lines, clonogenic capacity, which is regarded as a direct measure of stem cell function, was also reduced after treatment with birinapant in patient blast cells (Figure 2C). These results suggested that inhibition of XIAP might reduce cell viability and impair clonogenic capacity in AML cells.

### 3.3 XIAP inhibition induces apoptosis

**FIGURE 3:**
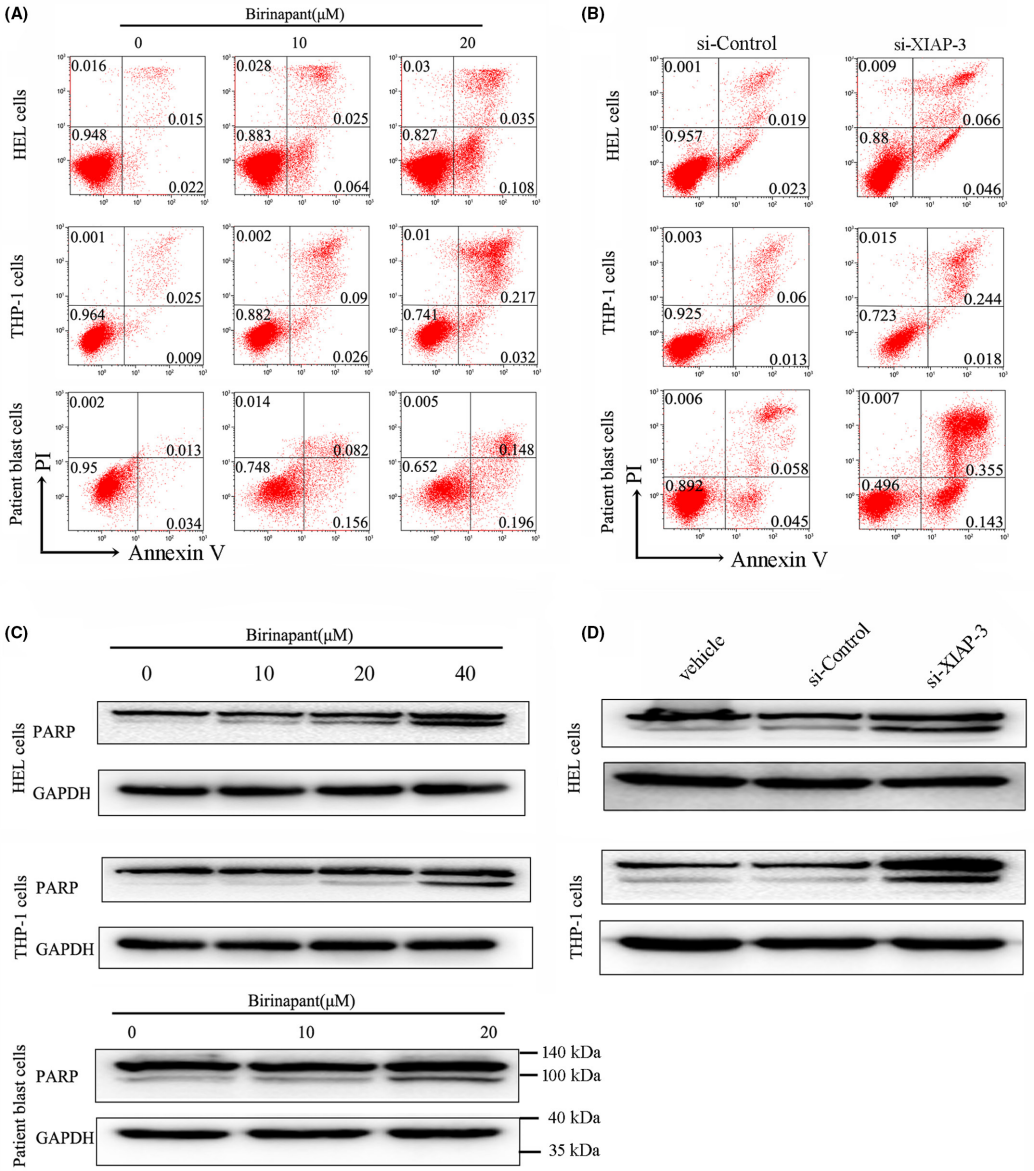
XIAP inhibition induces apoptosis in AML cells. (A, B) Two AML cell lines THP‐1 and HEL as well as patient blast cells were treated with various concentrations of birinapant or transfected with si‐XIAP‐3 for 48 h, and the apoptosis was determined by Annexin V‐FITC/PI. (C, D)The cleavage of PARP was also assessed for apoptosis in two AML cell lines and patient blast cells after treatment with birinapant or si‐XIAP‐3 for 48 h. Images representing four independent experiment were shown.

Then, we conducted flow cytometric analysis to clarify that birinapant dramatically induced apoptosis of AML cell lines as well as patient blast cells in a dose‐dependent manner (Figure 3A and Figure S3C). Similarly, the genetic inhibition of XIAP using siRNA also induced apoptosis in these AML cell lines and patient blast cells (Figure 3B). As expected, treatment with birinapant or XIAP siRNA promoted the cleavage of PARP, a classical marker of apoptosis, in THP‐1 and HEL cells as well as patient blast cells (Figure 3C,D and Figure S3D). These results suggested that XIAP inhibition might overcome the defects in apoptosis displayed by AML cells.

### 3.4 Autophagy induced by XIAP inhibition influences apoptosis and differentiation in AML cells

**FIGURE 4:**
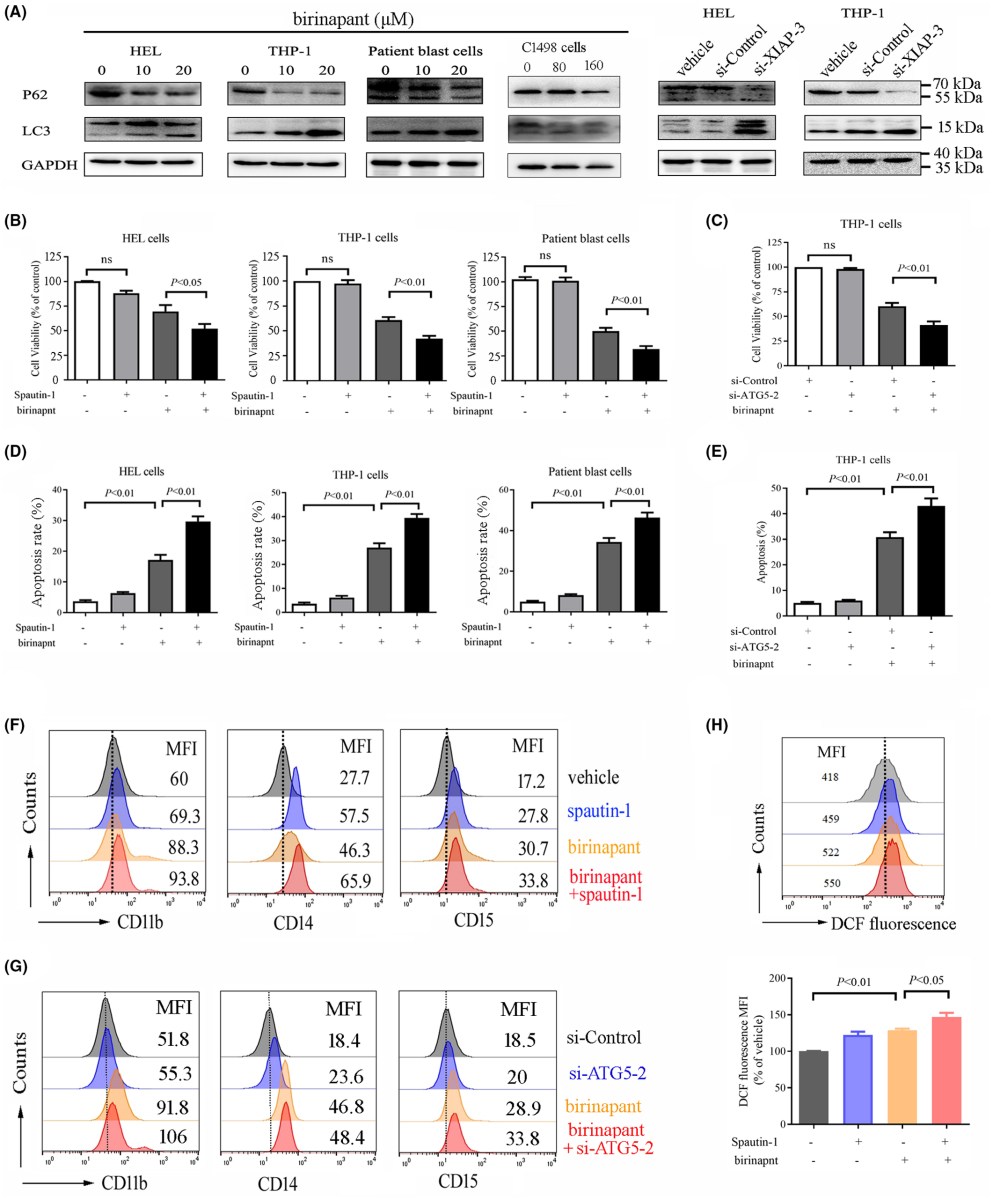
XIAP inhibition induces autophagy as a pro‐survival signal and inhibition of autophagy enhances the anti‐leukaemic effect of XIAP inhibition in AML cells. (A) AML cell lines HEL, THP‐1 and C1498 as well as patient blast cells were treated with various concentrations of birinapant or transfected with si‐XIAP for 48 h, and then, the autophagy markers LC3‐II and SQSTM1/p62 were determined by western blot analysis. (B, D) AML cells were treated with 20 μM birinapant, either alone or in combination with 10 μM spautin‐1 for 48 h, and subsequently their cell viability was determined using CCK‐8 assay, the apoptosis was assessed by flow cytometric analysis after staining with Annexin V/PI. (C, E) THP‐1 cells were transfected with si‐ATG5‐2 or si‐Control for 24 h, and subsequently treated either alone or in combination with 20 μM birinapant for 48 h, and then the cell viability and apoptosis were determined using CCK‐8 assay and flow cytometric analysis, respectively. (F) THP‐1 cells were treated with 20 μM birinapant, either alone or in combination with 10 μM spautin‐1 for 96 h, and subsequently evaluated for the differentiation by staining with CD11b, CD14 and CD15. Images shown were representatives of at least three independent experiments. (G) THP‐1 cells were transfected with si‐ATG5‐2 or si‐Control for 24 h, and subsequently treated either alone or in combination with 20 μM birinapant for 72 h, and then, the cells were collected and evaluated for the differentiation by staining with CD11b, CD14 and CD15. (H) THP‐1 cells were treated with 20 μM birinapant, either alone or in combination with 10 μM spautin‐1 for 6 h, and subsequently ROS level was determined using DCF‐DA assay with flow cytometric analysis, the top panel is the representative images, the bottom panel is the statistical data representing three independent experiments.

Mounting evidence indicates that XIAP promotes AML recurrence in caspase‐dependent inhibition of proteasome and apoptosis signalling pathways. Along with the deepening of research, XIAP has also been proposed to impact autophagy,^14^, ^16^ whereas hitherto the relationship between XIAP and autophagy is controversially discussed. To gain the specific role of XIAP on autophagy regulation in AML, we used birinapant or XIAP siRNA to treat AML cells and found that both birinapant and XIAP siRNA upregulated the levels of microtubule‐associated protein 1 light chain 3 (LC3‐II), a well‐established marker of autophagy, and consequently, downregulated the expression of SQSTM1/p62, an endogenous autophagy substrate, suggesting that XIAP may act as an inhibitor of autophagy and its inhibition can enhance autophagy in AML (Figure 4A, and Figure S4A). Then we used spautin‐1, a specific and potent autophagy inhibitor, combined with birinapant, and found that spautin‐1 and XIAP inhibitor synergistically impaired cell viability in all AML cells tested (Figure 4B). To further confirm the role of autophagy in the cytotoxicity of inhibition of XIAP, we examined the effect of silencing of ATG5, a key autophagy factor. We showed that siRNA‐mediated silencing of ATG5 (Figure S4B,C) enhanced the cytotoxicity of birinapant on THP‐1 cells (Figure 4C). Autophagy has a close relation with apoptosis. To investigate the effect of autophagy on apoptosis, we used birinapant with or without spautin‐1 to treat AML cells and found that combination with spautin‐1 enhanced apoptosis induced by birinapant (Figure 4D). Treatment with si‐ATG5‐2 also amplified the apoptotic effect induced by birinapant in THP‐1 cells (Figure 4E). These two results suggested that autophagy might act as a pro‐survival signal in XIAP inhibition. The blockade of cellular differentiation represents a central pathologic feature of AML and differentiation therapy has resulted in incredible cure rates in certain AML subtypes.^27^ We investigated the effect of autophagy induced by XIAP inhibition on myeloid cell differentiation, and found that combination with spautin‐1 enhanced the differentiation induced by birinapant, in which CD11b, CD14 and CD15 were significantly upregulated in THP‐1 cells (Figure 4F and Figure S4D). Similar results were also seen in treatment with si‐ATG5‐2 and birinapant in THP‐1 cells (Figure 4G and Figure S4E). The ROS pathway is deemed to closely correlate with differentiation and autophagy in AML cells. We further determined the ROS level in this condition, and found that combination with spautin‐1 upregulated the ROS level induced by birinapant (Figure 4H).

### 3.5 XIAP inhibition promotes autophagy via MDM2‐p53 pathway

**FIGURE 5:**
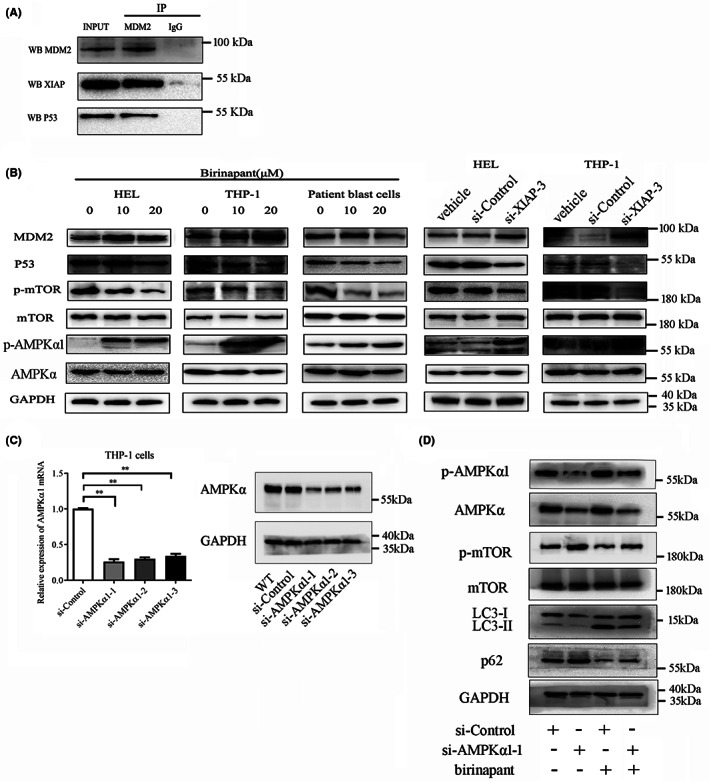
XIAP inhibition promotes autophagy through MDM2‐p53‐AMPK pathway. (A) Co‐IP showed that XIAP interacted with MDM2 and p53 in HEL cells. Ten percent whole cell lysate as INPUT samples was used. (B) XIAP inhibition using birinapant or si‐XIAP‐3 downregulated the level of p53 and the phosphorylation of mTOR, upregulated the phosphorylation of AMPK, but almost unaffected the level of MDM2 in AML cells. (C) siRNAs were designed to knockdown AMPKα1 and its effects were verified by relative expression of AMPKα1 mRNA and protein using qRT‐PCR and western blot in THP‐1 cells. (D) THP‐1 cells were transfected with si‐AMPKα1‐1 or si‐Control for 24 h, subsequently treated either alone or in combination with 20 μM birinapant for 48 h, and then, the cells were collected and determined for the expression of p‐AMPKα1, AMPKα1, p‐mTOR, mTOR, LC3‐I/II, p62 and GAPDH using western blot, respectively. Images shown were representatives of at least three independent experiments.

We previously reported that p53/AMPK/mTOR pathway could mediate autophagy in AML,^28^ while MDM2 is a negative regulator of p53.^14^ We further investigated the mechanism of autophagy induced by XIAP inhibition, and found that XIAP could interact with p53 and MDM2 in AML cells (Figure 5A). Thus, we explored whether XIAP functions through the p53/AMPK/mTOR signalling pathway. In THP‐1 and HEL cells as well as patient blast cells, inhibition of XIAP with birinapant or siRNA raised MDM2 expression while decreasing p53 expression, promoted the phosphorylation of AMPKα1, the predominant isoform of AMPK, while inhibiting mTOR phosphorylation (Figure 5B and Figure S5A). We silenced AMPKα1 using siRNA to further confirm the role of AMPKα1 in XIAP‐mediated effects on autophagy. Treatment with si‐AMPKα1‐1 (Figure 5C and Figure S5B) reduced the induction of autophagy by birinapant in THP‐1 cells, presenting with increased phosphorylation of mTOR, reduced conversion of LC3‐II from LC3‐I, and increased p62 (Figure 5D and Figure S5C). According to these findings, XIAP may control p53 via MDM2 and suppress autophagy via the AMPK/mTOR signalling pathway downstream of p53.

### 3.6 Autophagy inhibitor CQ retarded the AML progression in combination with XIAP inhibitor birinapant in vivo

**FIGURE 6:**
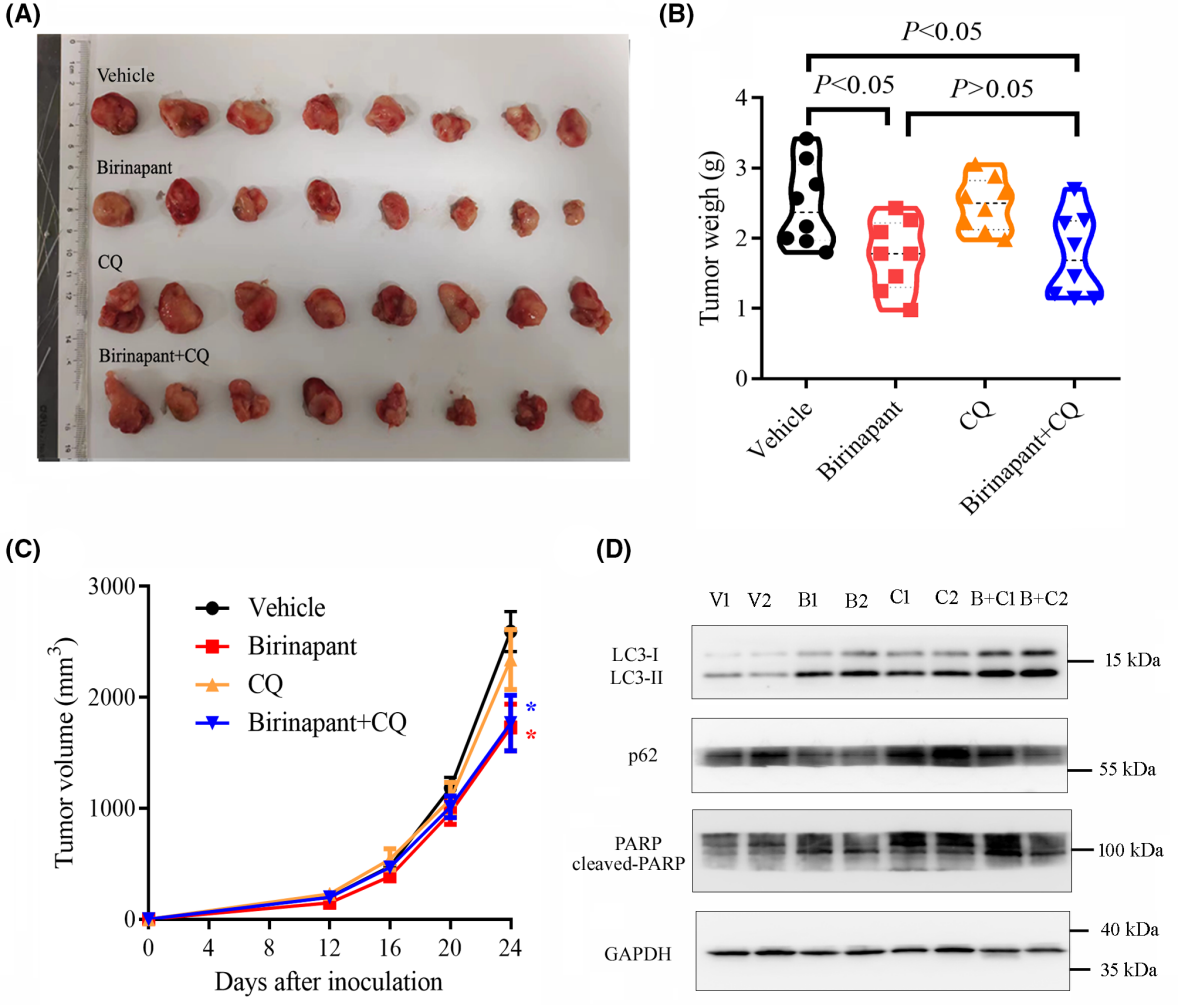
Autophagy inhibitor CQ had no significant effects on activity of birinapant in the xenograft model of HEL cells in NOD/SCID mice. (A, B) HEL cells (5 × 10^6^) were injected subcutaneously into the flank of female NOD/SCID mice (*n* = 8 for each group). When the tumour size reached about 150–200 mm^3^, intraperitoneal injections of birinapant (Bi; 40 mg/kg, three times per week) or chloroquine (CQ; 50 mg/kg, three times per week), or saline with 0.05% DMSO (Veh), or both Bi and CQ were administered for 14 days, and the size and weight of xenograft tumour were recorded after mice were killed. (C) Tumour volume was measured every 4 days. * represents *p* < 0.05 compared with the Veh treatment group. (D) The levels of LC3 and SQSTM1/p62, and the cleavage of PARP in tumour tissues were determined by western blot. Images shown were representatives of at least three independent experiments.

To investigate whether XIAP inhibition delayed leukaemia progression in vivo, we established a xenograft model in NOD/SCID mice by subcutaneous injection of HEL cells. After 2 weeks of treatment, tumour size and weight were obviously reduced in birinapant treatment group and combination treatment group compared to vehicle group, whereas there was no obvious difference in birinapant treatment group and combination treatment group (Figure 6A–C). Similar to in vitro experiments, birinapant elevated the LC3‐II level, accompanied by the down‐regulation of SQSTM1/p62 in tumour tissues in the birinapant treatment group (Figure 6D and Figure S6), indicating that autophagy does occur in the birinapant‐treated AML cells. The cleavage of PARP was more obviously observed in combination treatment group than birinapant treatment group (Figure 6D and Figure S6).

**FIGURE 7:**
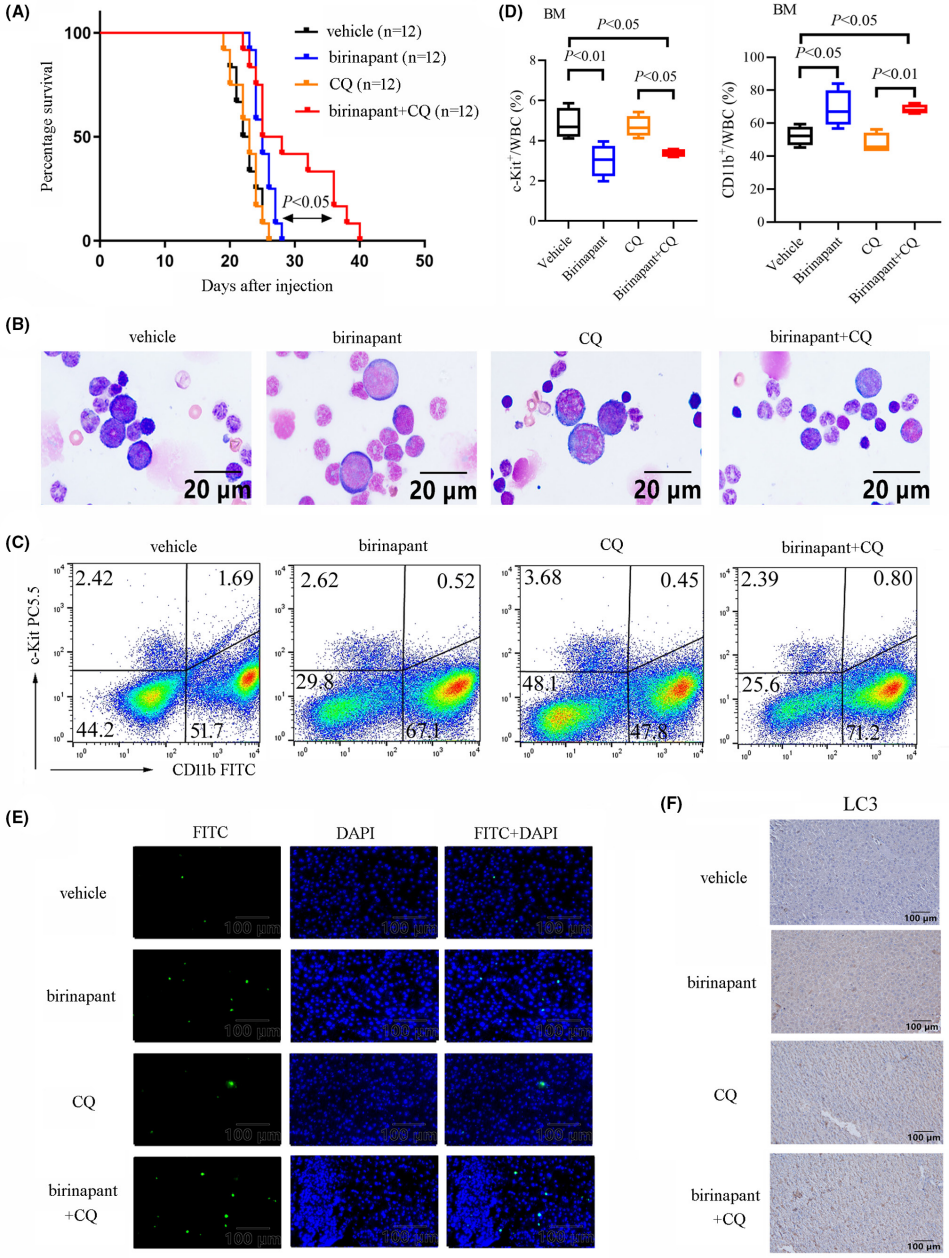
Autophagy inhibitor CQ enhances the antileukaemic effect of birinapant in vivo in C1498‐transplanted model. (A) C57BL/6 mice were injected intravenously with 1 × 10^6^ C1498 cells to establish orthotopic AML xenograft model (*N* = 16 mice per group). The Kaplan–Meier analysis with a Log‐rank test showed that combined treatment with birinapant and CQ had a longer survival than another three group. (B) Representative images of the Wright's staining of the BM aspirate smears were shown. (C, D) Representative images and statistical results of the proportion of c‐kit^+^ cells and CD11b^+^ cells in BM aspirates were shown on 15 days after transplantation. (E) Tunnel apoptotic analysis showed that combined treatment with birinapant and CQ and treatment with birinapant alone promoted apoptosis in liver tissue than another two group of mice. (F) The level of autophagy detected by LC3 using IHC was shown.

We also established an orthotopic xenograft model of AML in C57BL/6 mice to further validate the combination treatment strategy of XIAP inhibitor birinapant with autophagy inhibitor CQ for AML (Figure S7A). C1498 cells were injected via tail vein, following intraperitoneal administration of birinapant with or without CQ every 2 days. Interestingly, routine blood examination showed that there were no differences in various indicators among four groups (Figure S7B). Compared with the vehicle group, combination treatment with birinapant and CQ had a longer survival, whereas treatment with birinapant or CQ alone did not improve the survival of mice challenged with C1498 cells (Figure 7A). Wright's staining showed that combination treatment of birinapant and CQ decreased immature leukocytes and increased mature leukocytes in BM aspirates (Figure 7B). In line with these findings, flow cytometric analysis also found that the combination treatment group had a lower proportion of c‐kit^+^ cells and a higher proportion of CD11b^+^ cells in BM aspirates compared with those in vehicle group (Figure 7C,D). Combination treatment also increased the percentage of CD11b^+^ cells but had almost no impact on the proportion of c‐kit^+^ cells in PB compared with those in vehicle group (Figure S8A,B). Although there was no difference in the weight of the liver and spleen (Figure S7C,D) or the ratio of CD11b^+^ and c‐kit^+^ in liver (Figure S8C,D), the apoptotic analysis showed that combination treatment group had a higher level of apoptosis in liver tissues compared to vehicle group, whereas there was no significant difference between birinapant group and combination treatment group (Figure 7E). Similarly to apoptosis, XIAP inhibition upregulated the autophagic level detected by LC3 expression in liver tissues (Figure 7F). Above all, these results suggested that combination of XIAP inhibitor with autophagy inhibitor can retard the leukemogenesis and XIAP may be a potential therapeutic target.

## 4 DISCUSSION

Although a growing number of studies have demonstrated the role of XIAP in tumour initiation and progression, including haematological malignancies,^29^, ^30^ little is known about the prognostic implication of its expression in AML. In this study, we found that high expression of XIAP was an independent poor prognostic factor for AML patients. Meanwhile, we found that XIAP inhibition could upregulate the level of autophagy, which acts as a pro‐survival signal and an inhibitor of differentiation. Therefore, combination treatment of birinapant with CQ has a strong anti‐AML effect.

Previous studies concerning the relationship between the expression of XIAP and prognosis in solid cancers have shown convincing results.^31^, ^32^ Patients co‐expressing FOXM1, survivin, and nuclear XIAP had significantly worst OS in breast cancer.^32^ Especially, XIAP localized in nucleus, but not cytoplasm, is an independent prognostic factor in hormone receptor‐negative breast cancer patients.^3^ For AML, the prognostic significance of XIAP has been controversially discussed. Some studies showed that AML patients with higher XIAP protein expression underwent a markedly shorter survival and duration of remission and harboured intermediate or poor cytogenetics compared to patients with lower XIAP protein levels.^33^, ^34^, ^35^ On the contrary, a large study of 172 primary AML patient samples showed no correlation between XIAP protein expression and cytogenetics, remission achievement and OS.^36^ Another study showed that elevated protein, but not mRNA, level of XIAP was observed in childhood ALL cells compared with normal BMMNCs,^37^ suggesting that XIAP expression may be anomalously regulated post‐transcriptionally. Further molecular mechanism study had showed that XIAP harboured an internal ribosomal entry to engage alternative translation initiation during cellular stress conditions and finally maintain XIAP protein expression.^38^ In this present study, we found that in non‐M3 AML, patients with higher XIAP mRNA level had a worst OS compared with patients harbouring lower XIAP mRNA level. Multivariate analyses showed that besides XIAP expression, age and unfavourable karyotype were another independent prognostic factors for OS in AML patients.

XIAP, known for its key conserved inhibitory effects on caspase activity, functions as an inhibitor of cell apoptosis. Leu207 on the BIR2 domain of XIAP, strengthens its interaction with USP11, a deubiquitinase. Stabilisation of XIAP due to its deubiquitylation by USP11 results in the inhibition of apoptosis, which further facilitates tumorigenesis.^39^ Besides inhibiting apoptosis, XIAP has been shown to regulate inflammatory signalling, cell proliferation, autophagy as well as invasion and metastasis.^40^ In this current study, we focused on the role of XIAP in autophagy and interrelation of autophagy and apoptosis. We found that XIAP inhibition using pharmacologic or genetic knockdown dramatically impaired the cell viability and clonogenic capacity of AML cells through inducing apoptosis. XIAP inhibition also upregulated the level of autophagy, which acted as a pro‐survival signal. Treatment with birinapant also enhanced the level of ROS which promoting the myeloid cell differentiation of THP‐1 cells, and ROS has been reported to decrease the expression of XIAP,^29^, ^41^ suggesting that inhibition of XIAP may promote the differentiation of AML cells via upregulating ROS.

It has been shown that XIAP negatively regulates MDM2 stability through its ubiquitin E3 ligase activity, which suggests the existence of a feedback loop between XIAP and MDM2 under certain situation and uncovers the importance of autophagy inhibition in promoting tumorigenesis.^14^ Additionally, Liu et al. has identified the IRES region of XIAP mRNA that interacts with MDM2 protein, by which regulates the stabilisation of MDM2 via inhibiting its homodimerization.^42^ According to our present results, we believe that XIAP inhibition may decrease p53 via suppressing the degradation of MDM2 and upregulate autophagy via the AMPK/mTOR signalling pathway downstream of p53.

In addition to numerous studies expounding the mechanisms of anti‐apoptotic function of XIAP, a few studies have revealed that XIAP overexpression promotes colon cancer cell invasion via inhibiting Rho‐GDIα SUMOylation^43^ and facilitates bladder cancer invasion and lung metastasis via enhancing nucleolin‐mediated Rho‐GDIβ mRNA stability.^4^ Conversely, several reports also depicted XIAP as a tumour suppressor, among which a study revealed that caveolin‐1‐mediated XIAP recruiting to the α‐integrin complex could suppress cell migration and enhance cell adhesion.^44^ So, the role of XIAP as a driver or suppressor of metastasis may depend on the cancer tissue and cell type. In this study, although no obvious evidence showed that XIAP inhibitor birinapant could attenuate the liver metastasis, it obviously upregulated the apoptosis and autophagy level in liver tissues, which may be of great value to explore its anti‐leukaemia effect and potential clinical application in combination with conventional chemotherapy.

Even though XIAP was overexpressed in AML cells, the molecular mechanisms leading to XIAP upregulation remained unclear. Previous studies have showed that some transcription factor and microRNAs can regulate the XIAP expression in transcriptional or post‐transcriptional levels. miR‐200c has been reported to be downregulated, and in turn increased the stability of XIAP mRNA in bladder cancer.^45^ The FOXM1 transcription factor can bind the promoter region of XIAP and promote its transcription.^32^

In conclusion, we found that the expression of XIAP mRNA in BMMNCs was an independent prognostic factor for patients with AML. XIAP inhibition upregulated the autophagy level and autophagy inhibitor could aggravate the apoptosis and myeloid differentiation induced by XIAP inhibition in AML cells. And combination treatment of XIAP inhibition with autophagy inhibitor may serve as a promising therapeutic strategy against AML.

## AUTHOR CONTRIBUTIONS

**Ziyang Huang:** Investigation (equal); writing – original draft (equal). **Jifan Zhou:** Data curation (equal); investigation (equal). **Yinyan Jiang:** Formal analysis (equal); investigation (supporting); validation (equal). **Yixiang Han:** Conceptualization (equal); formal analysis (equal); supervision (equal). **Xiaofang Wang:** Formal analysis (equal). **Fanfan Li:** Formal analysis (equal). **Songfu Jiang:** Conceptualization (equal); supervision (equal). **Kang Yu:** Conceptualization (equal); funding acquisition (equal); supervision (equal). **Shenghui Zhang:** Conceptualization (equal); formal analysis (equal); funding acquisition (equal); writing – review and editing (equal).

## CONFLICT OF INTEREST STATEMENT

The authors declare no conflict of interest.

## Supporting information


Appendix S1

## Data Availability

Data sharing not applicable to this article as no datasets were generated or analysed during the current study.
