# Supplementary material for: Combined inhibition of XIAP and autophagy induces apoptosis and differentiation in acute myeloid leukaemia
Source: J Cell Mol Med. 2023 May 8;27(12):1682–96. doi: 10.1111/jcmm.17765 (PMC10273072; doi:10.1111/jcmm.17765)
Supplement: Supplementary file 1 — Appendix S1 [file JCMM-27-1682-s001.docx]

**Supplemental Figures with Figure legend**

**
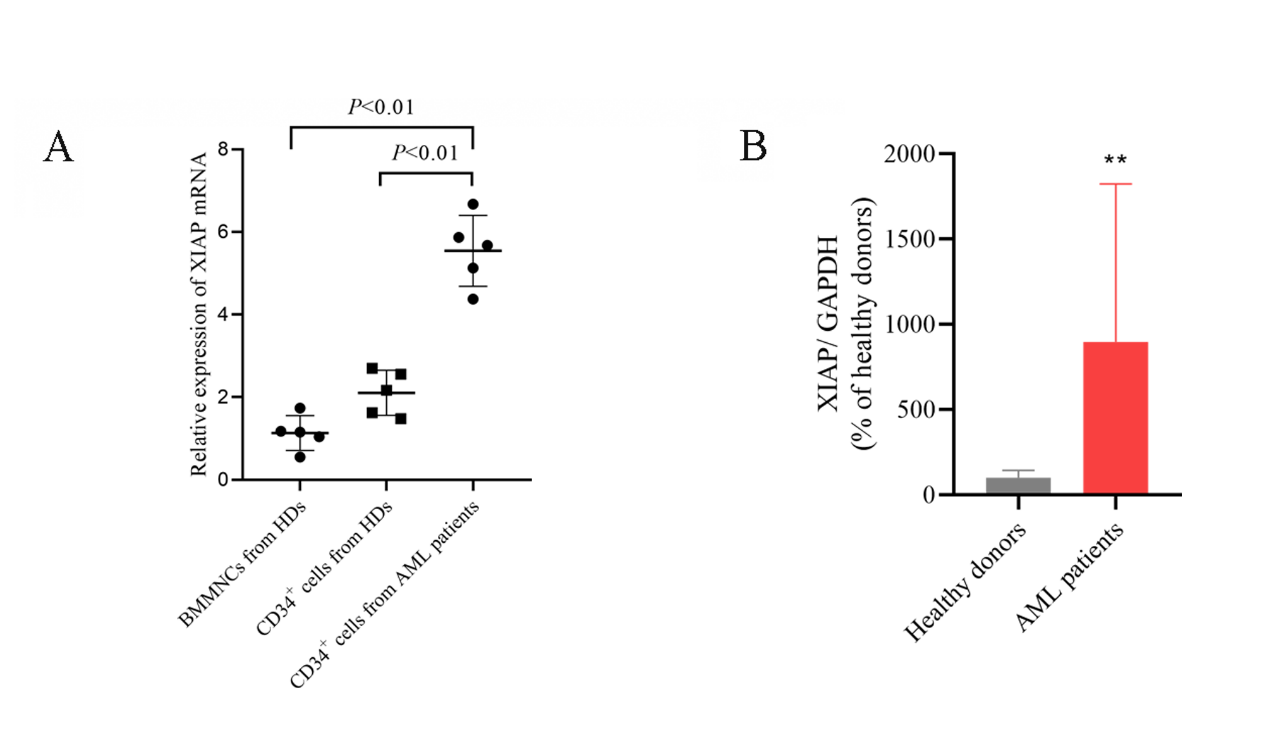
**

**Figure S1. XIAP was elevated in AML patients. (A)** Relative expression of XIAP mRNA was determined in BMMNCs from healthy donors (N=5), CD34^+^ cells from healthy donors (N=5) and CD34^+^ cells from newly diagnosed AML patients (N=5)**.** (**B**) The XIAP protein expression in BMMNCs from healthy donors (N=6) and newly diagnosed AML patients (N=33) was determined by western blot and the optical densities of the bands were analyzed using ImageJ software.


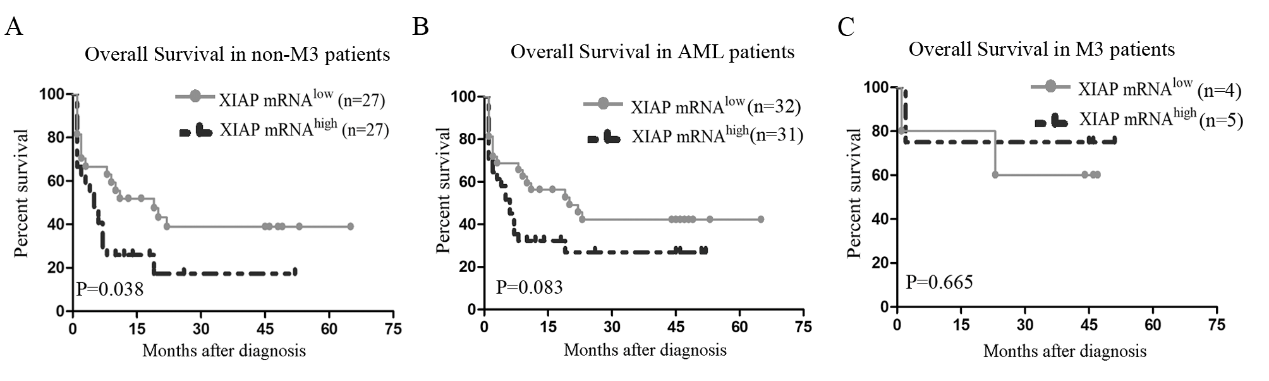


**Figure S2. The expression of XIAP mRNA was associated with overall survival in non-M3 AML patients.** (**A**) Patients with non-M3 AML with higher expression of XIAP mRNA had a shorter overall survival compared with those patients with lower expression of XIAP mRNA. (**B**) All AML patients with higher expression of XIAP mRNA had a trend to worse overall survival but no statistical difference compared with those patients with lower expression of XIAP mRNA. (**C**) APL Patients with higher expression of XIAP mRNA had no difference in overall survival compared with those patients with lower expression of XIAP mRNA.


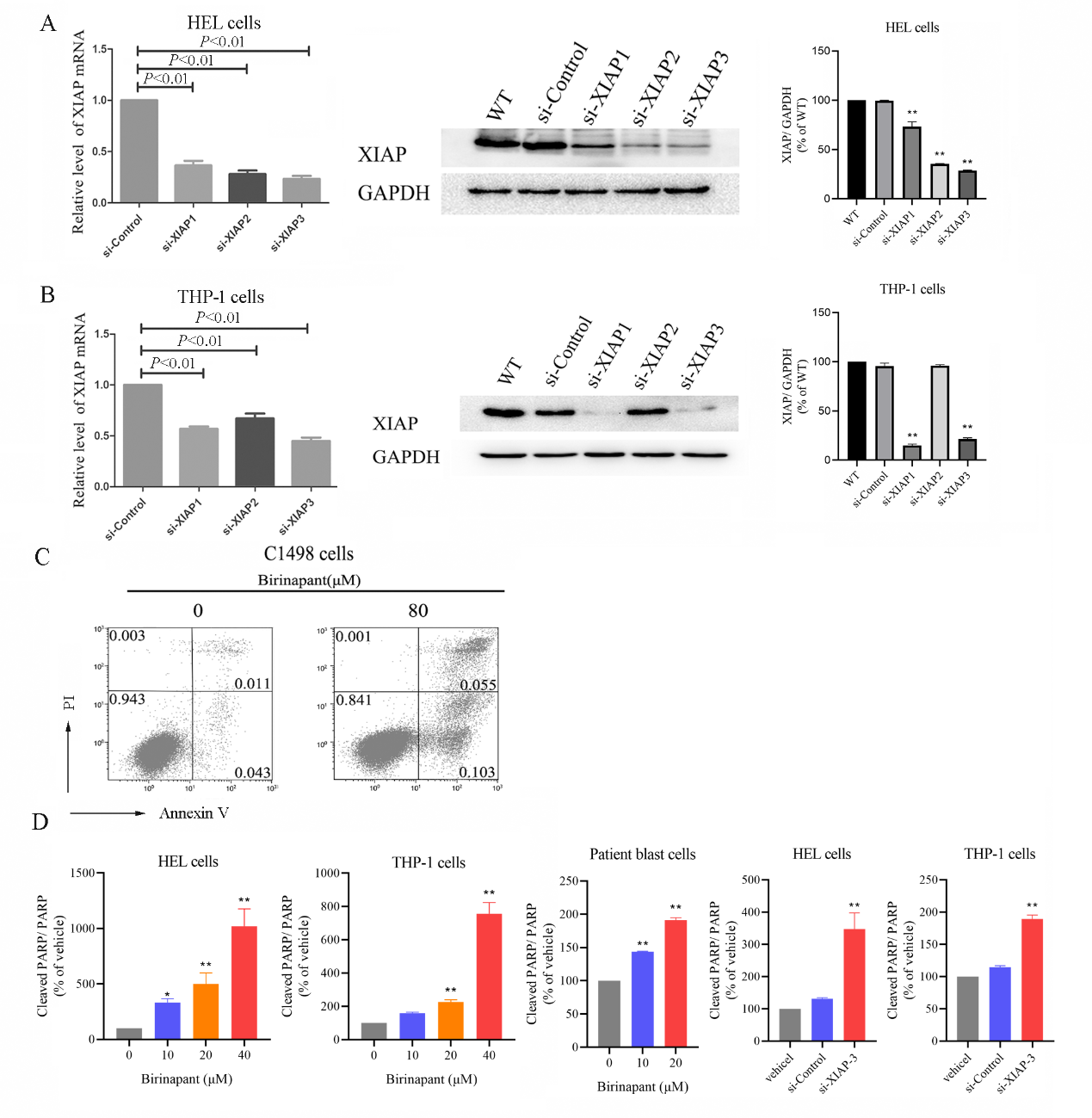


**Figure S3. Inhibition of XIAP induced apoptosis in AML cells.** (**A, B**) siRNAs were designed to knockdown XIAP and its effects were verified by relative expression of XIAP mRNA and protein using RT-PCR and western blot in HEL and THP-1 cells, and the optical densities of the bands were analyzed using ImageJ software. (**C**) Mouse AML cell line C1498 was treated with 0 or 80 µM birinapant for 48 hours, and the apoptosis was determined by Annexin V-FITC/PI with flow cytometric analysis. (**D**) Two AML cell lines THP-1 and HEL as well as patient blast cells were treated with various concentrations of birinapant or transfected with si-XIAP-3 for 48 hours, and the apoptosis marker cleaved PARP was determined by western blot and the optical densities of the bands were analyzed using Image J software. Data are expressed as mean ± SD representing at least three separate experiments. **p*<0.05, ***p*<0.01, vs. the respective control.


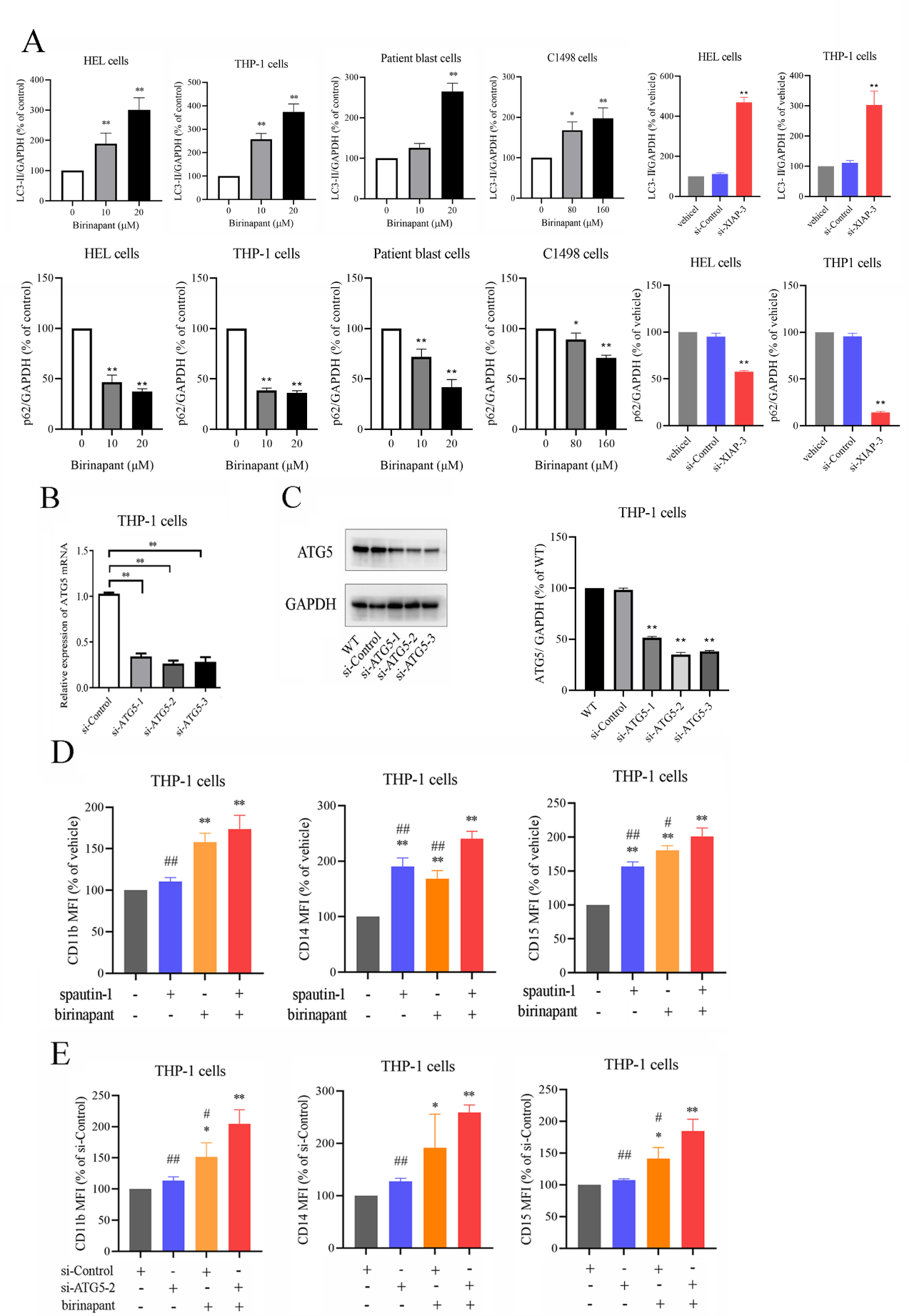


**Figure S4. XIAP inhibition induced autophagy and differentiation in AML cells.**  (A) AML cell lines HEL, THP-1, and C1498 as well as patient blast cells were treated with various concentrations of birinapant or transfected with si-XIAP-3 for 48 hours， and then, the autophagy markers LC3-II and SQSTM1/p62 were determined by western blot and the optical densities of the bands were analyzed using ImageJ software. (B, C) siRNAs were designed to knockdown ATG5 and that effects were verified by relative expression of ATG5 mRNA and protein using RT-PCR and western blot in THP-1 cells, and the optical densities of the bands were analyzed using ImageJ software. (D) THP-1 cells were treated with 20 μM birinapant, either alone or in combination with 10 μM spautin-1 for 96 hours, and then, THP-1 cells was collected and evaluated for the differentiation by staining with CD11b, CD14 and CD15. The mean fluorescence intensity (MFI) of CD11b, CD14, and CD15 was analyzed. (E) THP-1 cells were transfected with ATG5 siRNA-2 or si-Control for 24 hours, and subsequently treated either alone or in combination with 20 μM birinapant for 72 hours, and then, the cells were collected and evaluated for the differentiation by staining with CD11b, CD14 and CD15. Results are expressed as mean ± SD representing at least three independent experiments. **p*<0.05, ***p*<0.01, vs. the respective control. ^#^*p*<0.05, ^##^*p*<0.01, vs. the combined treatment group.


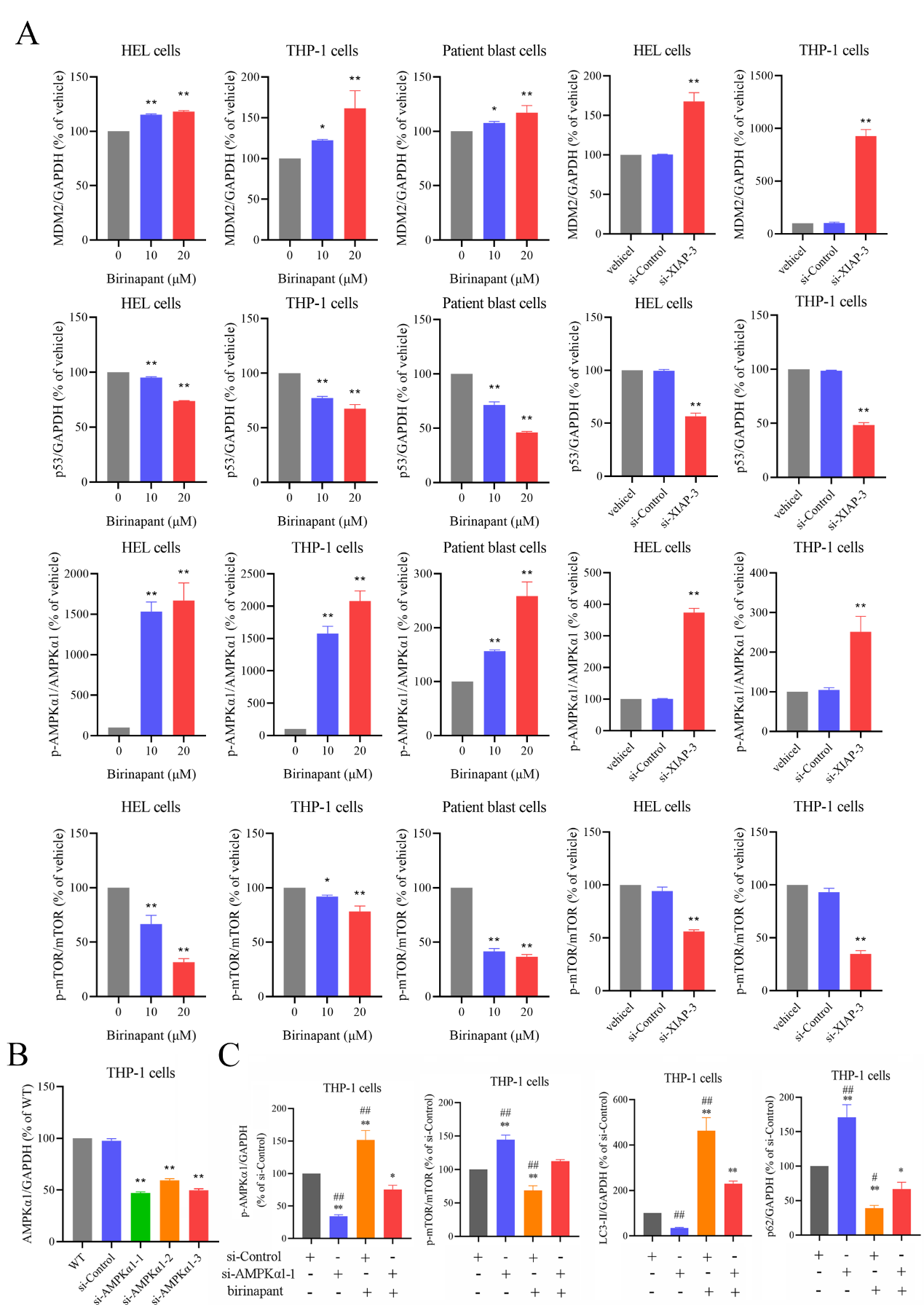


**Figure S5.** **XIAP inhibition promotes autophagy through MDM2-p53-AMPK pathway.** (**A**) AML cell lines HEL, THP-1, and C1498 as well as patient blast cells were treated with various concentrations of birinapant or transfected with si-XIAP-3 for 48 hours, and then, the cells were collected and determined for the expression of MDM2, p53, p-AMPKα1, AMPKα1, p-mTOR, mTOR and GAPDH using western blot, respectively. (B) siRNAs were designed to knockdown AMPKα1 and its effects were verified by expression of AMPKα1 protein using western blot in THP-1 cells and the optical densities of the bands were analyzed using ImageJ software. (C) THP-1 cells were transfected with si-AMPKα1-1 or si-Control for 24 hours, subsequently treated either alone or in combination with 20 μM birinapant for 48 hours, and then, the cells were collected and determined for the expression of p-AMPKα1, AMPKα1, p-mTOR, mTOR, LC3-I/II, p62 and GAPDH using western blot, respectively. Results are expressed as mean ± SD representing at least three independent experiments. **p*<0.05, ***p*<0.01, vs. the respective control. ^#^*p*<0.05, ^##^*p*<0.01, vs. the combined treatment group.


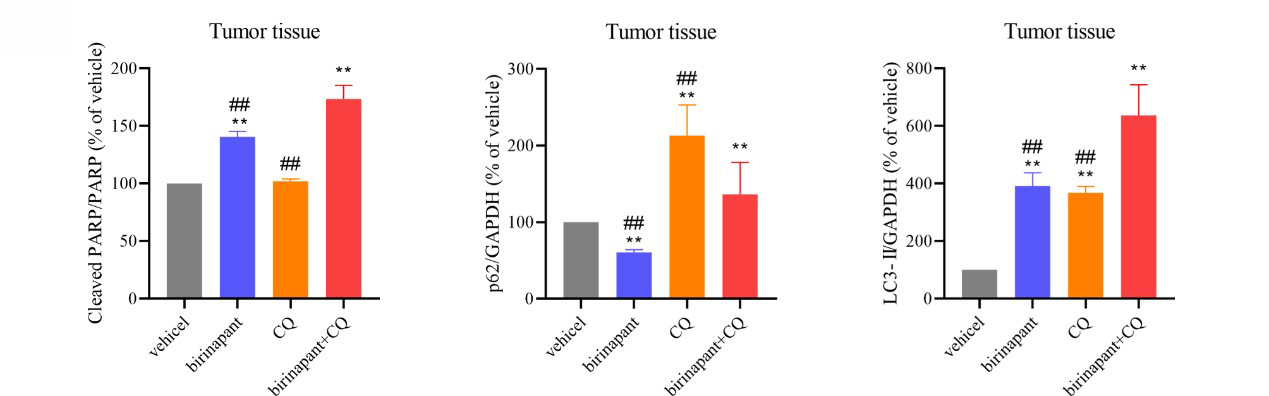


**Figure S6.** **XIAP inhibition elevated the levels of apoptosis and autophagy in tumor tissues and CQ enhanced XIAP inhibition-induced apoptosis.** The levels of LC3 and SQSTM1/p62, and the cleavage of PARP in tumor tissues were determined by western blot, and the optical densities of the bands were analyzed using ImageJ software. Results are expressed as mean ± SD representing eight tumor tissues each group. ^**^*P*<0.01, vs. the respective control. ^##^*P*<0.01, vs. the combined treatment group.


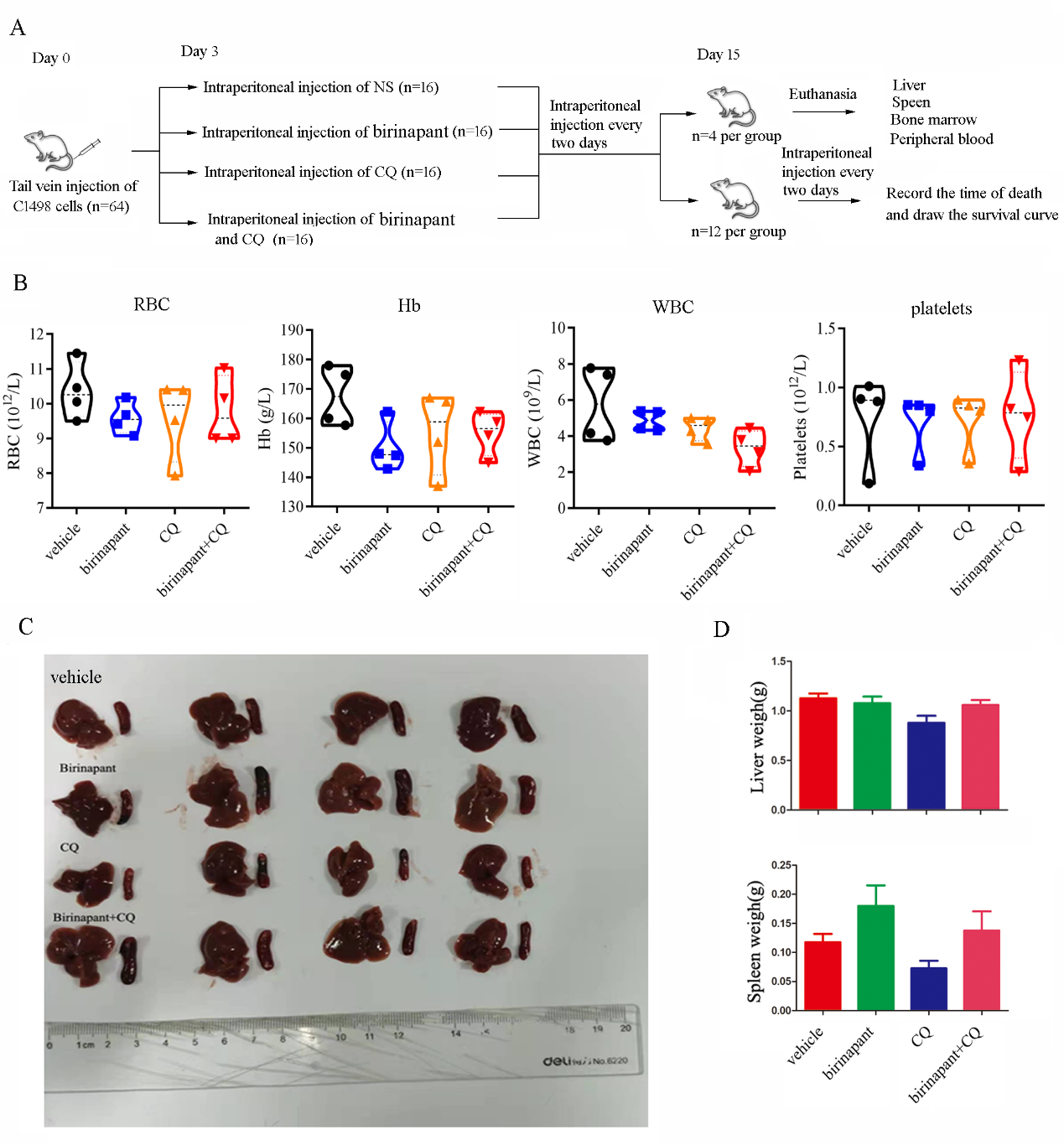


**Figure S7.** **The data of blood routine test and the weight of liver and spleen on 15 days after injection of C1498 cells in C57BL/6 mice.** (**A**) Experimental protocol was used to assess the anti-AML effect of the combination of birinapant with CQ on established orthotopic AML xenograft model. (**B**) Four mice per group were sacrificed under anesthesia on 15 days after tumor transplantation and quantitative data of RBC, Hb, WBC, and PLT in peripheral blood was presented in bar charts. (**C, D**) Spleen and liver of 4 mice per group on 15 days after tumor transplantation were presented, and the weight of liver (top label) and spleen (bottom label) were shown.


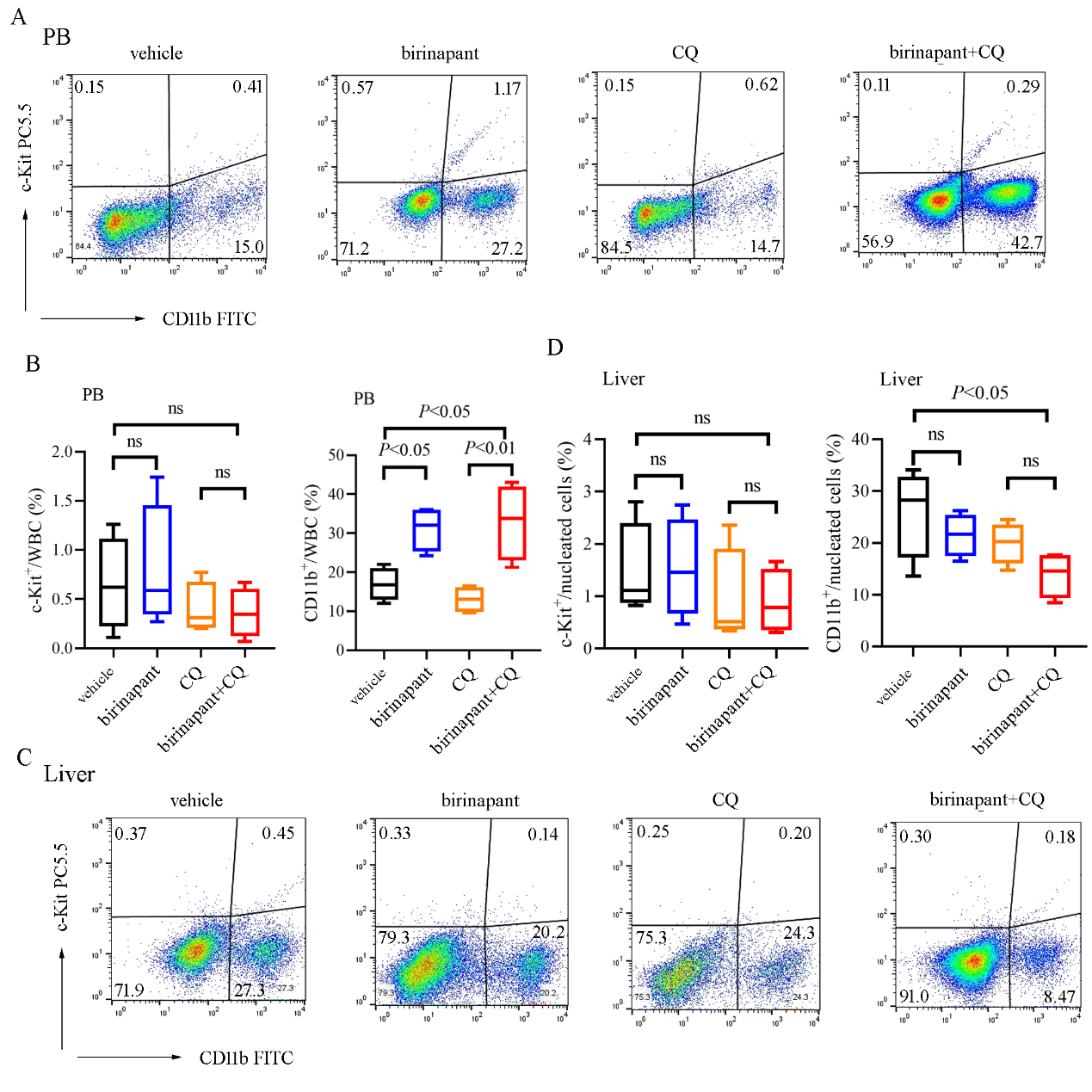


**Figure S8. Combined inhibition of XIAP and autophagy promoted the differentiation and lessened liver metastasis on 15 days after injection of C1498 cells in C57BL/6 mice.** (**A, B**) Representative images and statistical results of the proportion of c-kit^+^ cells and CD11b^+^ cells in peripheral blood were shown. (**C, D**) Representative images and statistical results of the proportion of c-kit^+^ cells and CD11b^+^ cells in liver tissues were shown.

| **Table S1.** Expression of XIAP mRNA in 63 AML patients. | | | | | |  |
| --- | --- | --- | --- | --- | --- | --- |
| Patient no. | XIAP mRNA expression | Molecular cytogenetic aberration | Cytogenetic abnormalities | Fab Type | Risk stratification | Enrolled date |
| 1 | 4.044608323 | FLT3-ITD | 46,XX | M4 | Adverse | 2016/8/20 |
| 2 | 2.228387302 | WT1 | 46,XX | M5 | Intermediate | 2017/1/18 |
| 3 | 2.259494429 | PML-RARA | complex chromosomal aberrations | M3 | APL | 2017/2/14 |
| 4 | 1.185914499 | WT1 | complex chromosomal aberrations | M5 | Adverse | 2017/3/18 |
| 5 | 2.801112642 | - | 46,XY | M5 | Intermediate | 2017/6/13 |
| 6 | 4.453686473 | WT1 | 46,XY | M4 | Intermediate | 2017/9/1 |
| 7 | 2.388326374 | - | 46,XX | M5 | Intermediate | 2017/11/20 |
| 8 | 3.988925005 | - | 46,XY | M2 | Intermediate | 2017/11/28 |
| 9 | 2.650014034 | NPM1, DNMT3A, TET2, FLT3-ITD | 46,XX | M4 | Intermediate | 2017/12/6 |
| 10 | 1.032398535 | - | 46,XX | M4 | Intermediate | 2018/1/30 |
| 11 | 14.78436116 | PML-RARA | 46,XX 15,17 | M3 | APL | 2018/2/4 |
| 12 | 0.294430697 | - | 46,XX | M4 | Intermediate | 2018/2/27 |
| 13 | 0.820172911 | - | 46,XX | M4 | Intermediate | 2018/3/3 |
| 14 | 0.807760778 | WT1 | complex chromosomal aberrations | M4 | Adverse | 2018/3/3 |
| 15 | 0.856188285 | - | 46,XY | M4 | Intermediate | 2018/3/4 |
| 16 | 3.47256091 | - | complex chromosomal aberrations | M5 | Adverse | 2018/3/8 |
| 17 | 4.269301352 | PML-RARA | 46,XY | M3 | APL | 2018/3/12 |
| 18 | 0.782411782 | NARS, FLT3-TKD, PML-RARA | 46XY 15,17 | M3 | APL | 2018/3/18 |
| 19 | 0.240815474 | - | 46,XY | M2 | Intermediate | 2018/4/2 |
| 20 | 2.661058082 | PML-RARA | 46,XX | M3 | APL | 2018/4/4 |
| 21 | 29.77438905 | CEBPA++, IDH2, WT1 | 47,XX,+8 | M4 | Intermediate | 2018/4/8 |
| 22 | 1.72428709 | MLL-9 | 46,XX | M5 | Intermediate | 2018/4/10 |
| 23 | 1.046810282 | BCR-ABL | complex chromosomal aberrations | M5 | Adverse | 2018/4/13 |
| 24 | 3.285242803 | CEBPA++, IDH2 | 46,XX | M4 | Favorable | 2018/4/19 |
| 25 | 2.879862637 | PML-RARA | 46XX, 15,17 | M3 | APL | 2018/4/20 |
| 26 | 3.129647127 | - | 46,XY | M4 | Intermediate | 2018/4/22 |
| 27 | 1.039579435 | IDH1\ASAL1\TET2\TP53 | 46,XY | M2 | Adverse | 2018/4/23 |
| 28 | 7.205007839 | NARS | 47,XY，+8 | M5 | Intermediate | 2018/5/7 |
| 29 | 2.455471368 | PHF\ASXL4 | 46,XY | M5 | Intermediate | 2018/5/7 |
| 30 | 1.748357241 | WT1, FLT3-ITD | 46,XY | M5 | Adverse | 2018/5/11 |
| 31 | 0.512633619 | - | 46,XY | M5 | Intermediate | 2018/5/14 |
| 32 | 3.47256091 | NARS, BCOR | 46,XY | M5 | Intermediate | 2018/5/24 |
| 33 | 1.121943481 | TET2, FLT3-ITD | 46，XX，t（6,13） | M5 | Adverse | 2018/5/28 |
| 34 | 1.106497353 | - | 45X,-X,t（8：:21） | M4 | Intermediate | 2018/5/28 |
| 35 | 0.873572896 | - | 46,XX | M4 | Intermediate | 2018/6/3 |
| 36 | 1.842928372 | WT1 | 46XX，inv16 | M4 | Favorable | 2018/6/12 |
| 37 | 1.362258035 | - | 46,XY | M4 | Intermediate | 2018/6/21 |
| 38 | 1.52414483 | NPM1, IDH2 | 46,XX | M5 | Favorable | 2018/6/25 |
| 39 | 2.200757219 | PML-RARA | 46,XX | M3 | APL | 2018/6/28 |
| 40 | 1.460032011 | PML-RARA | 46XY 15,17 | M3 | APL | 2018/7/12 |
| 41 | 1.72428709 | PML-RARA | complex chromosomal aberrations | M3 | APL | 2018/7/16 |
| 42 | 0.719965659 | NARS, WT1, CBFB-MYH11 | complex chromosomal aberrations | M4 | Adverse | 2018/8/4 |
| 43 | 2.008335086 | - | 46,XY | M5 | Intermediate | 2018/8/16 |
| 44 | 5.300028068 | MLL-AF9, TP53 | 47xx，+8 | M5 | Adverse | 2018/8/26 |
| 45 | 2.807327254 | DNMT3A, ASXL1, FLT3-ITD, DDFREYE, NPM1 | 46,XX | M5 | Adverse | 2020/4/26 |
| 46 | 2.052927966 | WT1\KRAS, BCOR | 46，XX，t（10,11） | M5 | Adverse | 2020/6/23 |
| 47 | 5.498366093 | WT1, SF3B1, BCOR | 46，XX，t（3,3） | M5 | Adverse | 2020/8/7 |
| 48 | 3.528194623 | DNMT3A, TET2, FLT3 | 46,XX | M5 | Adverse | 2020/8/21 |
| 49 | 3.131994867 | WT1 | 46XY INV（9） | M5 | Intermediate | 2020/8/25 |
| 50 | 4.152975418 | nars\ASXL1\EZH2 | 47XY +19 | M5 | Adverse | 2020/9/21 |
| 51 | 2.455107808 | GATA2, WT1, NPM1 | 46,XX | M4 | Favorable | 2020/9/25 |
| 52 | 3.555526821 | NARS, WT1, CBFB-MYH11 | 46,XY | M4 | Favorable | 2020/11/12 |
| 53 | 3.156082534 | CEBPA++, WT1 | 46,XY | M5 | Favorable | 2020/11/23 |
| 54 | 3.391421103 | KARS, NARS, FLT3-ITD | 47,XX，+8 | M4 | Adverse | 2021/1/25 |
| 55 | 3.293264029 | CEBPA++, WT1 | 46,XX | M5 | Favorable | 2021/2/17 |
| 56 | 3.103711747 | CEBPA+, WT1,FLT3-ITD | 46,XY | M4 | Adverse | 2021/2/23 |
| 57 | 12.55022247 | MLL-AF6, 10, EXL, ELL\WT1 | complex chromosomal aberrations | M5 | Adverse | 2021/2/26 |
| 58 | 5.552374773 | TET1\TET2\NPM1\FLT3-ITD\WT1 | 46,XX | M5 | Intermediate | 2021/2/26 |
| 59 | 2.356801462 | WT1, CEBPA\IDH2 | 46,XY | M5 | Intermediate | 2021/3/20 |
| 60 | 2.824198229 | RUNX1\WT1 | 46xy der（17,18） | M5 | Adverse | 2021/3/24 |
| 61 | 6.998568016 | NPM1\TET2\FLT3-ITD, WT1 | 46,XY | M5 | Intermediate | 2021/4/10 |
| 62 | 4.17141227 | WT1, DNMT3A, NPM1, FLT3-ITD | 46,XY | M5 | Intermediate | 2021/4/19 |
| 63 | 4.778958437 | WT1\CEBPA+\NPM1\TET2\FLT3-ITD | 46,XY | M4 | Intermediate | 2021/4/20 |

| **Table S2.** Sequences of the primers used for real-time PCR. | | |
| --- | --- | --- |
| Genes | Forward primer (5’-3’) | Reverse primer (5’-3’) |
| *XIAP* | GCAGAGCGGAGTTGGCATT | CACCTTTTCGCGCCAGGAC |
| *ATG5* | AAGCTGTTTCGTCCTGTGGC | TCAATCTGTTGGCTGTGGGATG |
| *AMPKα1* | ATGCGCAGACTCAGTTCCTG | CTTCACTTTGCCGAAGGTGC |
| *GAPDH* | ATCATCAGCAATGCCTCC | CATCACGCCACAGTTTCC |

XIAP, X-linked inhibitor of apoptosis protein

ATG5, autophagy related 5

AMPKα1, AMP-activated protein kinase, alpha 1 catalytic subunit

GAPDH, glyceraldehyde 3-phosphate dehydrogenase

**Table S3.** The sequences of XIAP siRNAs, ATG5 siRNAs and AMPKα1 siRNAs.

| Name | sequences (5’-3’) | | |
| --- | --- | --- | --- |
|  | sense | antisense | |
| si-Control | UUCUCCGAACGUGUCACGUTT | | ACGUGACACGUUCGGAGAATT |
| si-XIAP-1 | GGUCAGUACAAAGUUGAAATT | | UUUCAACUUUGUACUGACCTT |
| si-XIAP-2 | CAUGGAUAUACUCAGUUAATT | | UUAACUGAGUAUAUCCAUGTT |
| si-XIAP-3 | CCAUGUGCUACACAGUCAUTT | | AUGACUGUGUAGCACAUGGTT |
| si-ATG5-1 | GUGAUGAUUCAUGGAAUUGTT | | CAAUUCCAUGAAUCAUCACTT |
| si-ATG5-2 | GCAGUGGCUGAGUGAACAUTT | | AUGUUCACUCAGCCACUGCTT |
| si-ATG5-3 | GCUAGCUGGCUGUCCAUAUTT | | AUAUGGACAGCCAGCUAGCTT |
| si-AMPKα1-1 | GCAGAAGUAUGUAGAGCAAUCTT | | GAUUGCUCUACAUACUUCUGCTT |
| si-AMPKα1-2 | GCUUGAUGCACACAUGAAUTT | | AUUCAUGUGUGCAUCAAGCTT |
| si-AMPKα1-3 | CCUUUCUGGUGUGGAUUAUTT | | AUAAUCCACACCAGAAAGGTT |

XIAP, X-linked inhibitor of apoptosis protein

ATG5, autophagy related 5

AMPKα1, AMP-activated protein kinase, alpha 1 catalytic subunit

**Table S4.** Comparison of clinical manifestations and laboratory features between patients with non-M3 AML with lower or higher XIAP expression.

| **Variable** | | **Whole cohort (n=54)** | | **Higher XIAP expression (n=27)** | | | **Lower XIAP expression (n=27)** | | | **P** |  |  |
| --- | --- | --- | --- | --- | --- | --- | --- | --- | --- | --- | --- | --- |
| WT1 | 23 | | 15 | | | 8 | | | 0.0820 | | | |
| FLT3-ITD | 15 | | 8 | | | 6 | | | 0.7570 | | | |
| CEBPA++ | 5 | | 4 | | | 1 | | | 0.3475 | | | |
| NPM1 | 11 | | 7 | | | 4 | | | 0.4936 | | | |
| TET2 | 5 | | 3 | | | 2 | | | 1 | | | |
| DNMT3A | 6 | | 4 | | 2 | | | 0.6662 | | | |  |
| Unfavorable Karyotype | 16 | | 9 | | 7 | | | 0.7664 | | | |  |
| Advanced | 25 | | 15 | | 10 | | | 0.2749 | | | |  |
| Age (years) | 53 (15-84) | | 50 (16-84) | | 54 (15-77) | | | 0.8285 | | | |  |
| WBC (/mL) | 25.28 (0.86-491.53) | | 23.5 (0.86-362.58) | | 32.66(1.63-491.53) | | | 0.1945 | | | |  |
| neutrophils(/mL) | 0.26 (0-19.8) | | 0.42 (0-19.8) | | 0.09 (0-0.72) | | | 0.0005 | | | |  |
| Platelets (×10^3^/mL) | 55.5 (5-912) | | 63 (5-912) | | 51 (10-231) | | | 0.7489 | | | |  |
| Hemoglobin (g/dL) | 85.5 (26-146) | | 96(39-131) | | 83(26-146) | | | 0.4319 | | | |  |
| Blast (%) | 41(0-96) | | 36(0-95) | | 47(0-96) | | | 0.2192 | | | |  |

**Table S5.** Univariate analysis of the effects of clinical parameters, BM XIAP expression, and molecular alterations on overall survival in patients with non-AML.

| **Variable No. of patients *P* Vaule** | | |
| --- | --- | --- |
| **Age,year** |  | 0.0172 |
| ≥60 | 22 |  |
| <60 | 32 |  |
| **Sex** |  | 0.4308 |
| Male | 29 |  |
| Female | 25 |  |
| **WBC** |  | 0.8895 |
| ＞50000/μl | 18 |  |
| ≤50000/μl | 36 |  |
| **PLT** |  | 0.2011 |
| ≤100000/μl | 36 |  |
| ＞100000/μl | 18 |  |
| **Hb** |  | 0.2659 |
| ≤100g/L | 42 |  |
| ＞100g/L | 12 |  |
| **Blast** |  | 0.6745 |
| ≥20% | 42 |  |
| ＜20% | 12 |  |
| **Karyotype** |  | 0.1671 |
| Favorable | 8 |  |
| Intermediate | 27 |  |
| Unfavorable | 19 |  |
| **Chromosome** |  | 0.0944 |
| Normal | 38 |  |
| Abnormal | 16 |  |
| **WT1 expression (>2.5 × 10^-2^)** |  | 0.6978 |
| positive | 23 |  |
| negative | 31 |  |
| **FLT3-ITD** |  | 0.2360 |
| Mutated | 14 |  |
| Wild | 40 |  |
| **CEBPA** |  | 0.2963 |
| Double mutation | 5 |  |
| Others | 49 |  |
| **NPM1** |  | 0.5805 |
| Mutated | 11 |  |
| Wild | 43 |  |
| **TET2** |  | 0.0978 |
| Mutated | 5 |  |
| Wild | 49 |  |
| **DNMT3A** |  | 0.8459 |
| Mutated | 6 |  |
| Wild | 48 |  |

**Table S6.** Multivariate analysis (Multiple Linear Regression) on the overall survival in patients with AML.

| **Variable** | ***t*** | ***P* vaule** |
| --- | --- | --- |
| Age^*^ | -3.065 | 0.004 |
| XIAP^♯^ | -3.046 | 0.004 |
| Unfavorable karyotype^Δ^ | -2.584 | 0.013 |
| TET2^¶^ | -0.065 | 0.518 |

^*^Age older than 60y relative to age 60 or younger.

^♯^High XIAP expression vs. lower XIAP expression.

^Δ^Unfavorable karyotype vs. other karyotype.

^¶^ TET2 mutation vs. other subtypes.
